# Prediction of antimicrobial resistance in *Staphylococcus aureus* with a machine learning classifier based on WGS data

**DOI:** 10.1128/spectrum.00065-25

**Published:** 2025-08-05

**Authors:** Ying Liu, Xudong Wang, Liangquan Wu, Zhenzhen Shi, Miao Zhu, Linhui Ye, Ping Xu

**Affiliations:** 1 Department of Tuberculosis, The Second Hospital of Nanjing, Nanjing University of Chinese Medicine 531909, Nanjing, China; 2 Department of Critical Care Medicine, the Affiliated Xuzhou Municipal Hospital of Xuzhou Medical University, Xuzhou, China; 3 Department of Critical Care Medicine, Xuzhou First People’s Hospital, Xuzhou, China; 4 Department of Respiratory and Critical Care Medicine, The Affiliated Jiangning Hospital of Nanjing Medical University 579164 , Nanjing, China; 5 Dinfectome Inc. 651565 , Nanjing, China; 6 Medical Department, Nanjing Lishui People's Hospital, Zhongda Hospital, Lishui Branch, Southeast University 670176 , Nanjing, China; 7 Department of Clinical Laboratory, The Fifth People's Hospital of Suzhou 590441, Suzhou, China; Universita degli Studi dell'Insubria, Varese, Italy

**Keywords:** rapid antimicrobial susceptibility testing, antimicrobial resistance, WGS, machine learning, *Staphylococcus aureus*

## Abstract

The phenomenon of antimicrobial resistance (AMR) often results in treatment failure and restrictions on precision medicine, emphasizing the need for molecular diagnosis of drug resistance. The current use of machine learning (ML) techniques based on whole genome sequencing (WGS) data offers a more precise prediction of phenotypes. We incorporated WGS data from 3979 *Staphylococcus aureus* strains in our study. We modeled 10 common antibiotics using three types of features: gene, single nucleotide polymorphism (SNP), and k-mer to identify the best model and to determine which feature values most significantly contributed to the model’s performance. The area under the curve (AUC) values of 40 mL models for 10 antibiotics ranged from 0.8345 to 0.9995. We noted that the performance indices such as the AUC of the gene model (0.9311–0.9992) and the integrated model (0.9313–0.9995) were markedly better than the SNP model (0.8345–0.9933) and the k-mer model (0.9024–0.9969). The best model AUC values for six antibiotics—cefoxitin, tetracycline, methicillin, gentamicin, erythromycin, and clindamycin—were over 0.99; nine antibiotic models had AUC values over 0.96, and all could effectively predict AMR phenotypes. Additionally, we discovered that certain non-AMR genes, such as the X998_03220 gene, significantly contributed to drug resistance prediction and overlapped in various antibiotic-related models simultaneously. Our study developed ML models that can reliably predict AMR phenotypes for commonly used antibiotics in *S. aureus*. We also identified potential molecular markers that can contribute to precision medicine implementation and healthcare cost reduction.

**IMPORTANCE:**

In our study, we developed a machine learning (ML) model that reliably predicts the antimicrobial resistance (AMR) phenotypes of *Staphylococcus aureus* to commonly used antibiotics. This model not only predicts AMR phenotypes but also identifies potential molecular markers, which could facilitate the implementation of precision medicine and contribute to reducing healthcare costs. The integration of diverse biomarker types is crucial for enhancing model performance; however, their effectiveness may vary depending on the specific antibiotic in question. Furthermore, our pan-genome-based characterization has revealed novel potential molecular markers associated with AMR, thereby enhancing our comprehension of the underlying molecular mechanisms of AMR in *S. aureus*. The expedited implementation of early and targeted antimicrobial therapies for *S. aureus* infections is essential for advancing precision medicine and can potentially lead to significant healthcare cost savings.

## INTRODUCTION

The introduction and use of antibiotics in the early 20th century revolutionized traditional medical treatment and became a critical component of modern healthcare (1). However, the rising issue of antimicrobial resistance (AMR) has notably affected the effectiveness of these treatments, resulting in worse patient outcomes and increased financial burden (2). In early 2022, the Antimicrobial Resistance Collaborators reported that in 2019, there were roughly 4.95 million deaths related to AMR, with 1.27 million directly linked to bacterial AMR. Furthermore, the study identified *Staphylococcus aureus*, among other pathogens, as a significant contributor to AMR-related deaths (3).

In order to circumvent the inefficiency of antibiotic treatment, the clinical choice of antibiotic is primarily made through an antimicrobial susceptibility test (AST). However, even for fast-growing bacteria, AST requires a minimum of 2 days to complete, and the validation period for slower-growing microorganisms is significantly longer (4). Regrettably, AST still relies on culture, a delay that has contributed to the proliferation of AMR (5). With the advancement of sequencing technology, a deeper understanding of the correlation between genotype and AMR phenotype has been established (6), which has prompted the establishment of machine learning (ML)-based AMR phenotype prediction models. Numerous studies have demonstrated that for certain bacteria, AMR phenotypes can be accurately predicted using data obtained from whole genome sequencing (WGS) (4, 7, 8).

In the early stages, models predicting AMR phenotypes typically incorporate antibiotic resistance genes (ARGs) as input features. For instance, Van Camp et al. (9) utilized known ARGs to construct an ML model with high accuracy and processing speed for AMR phenotype prediction, which could expedite clinical decision-making. Similarly, Yang and Wu (10) forecasted AMR phenotypes in four microorganisms, including *S. aureus*, by creating pan-genome data sets and extracting gene presence/absence patterns, achieving the model receiver operating characteristic (ROC) value exceeding 0.95. Currently, rapid molecular testing forms for resistance prediction have been integrated into clinical practice where the genotype-phenotype relationship is simple, such as testing the mecA gene to determine if *S. aureus* is methicillin-resistant (11). However, AMR is complex, and in many cases, genotypes and phenotypes do not align perfectly, particularly across multiple species or antibiotics. Consequently, relying solely on gene-level data, especially known ARGs, could lead to prediction errors due to poor characterization, such as missing gene data for different exocytosis mechanisms (12).

In addition, only reliance on ARGs may lead to false negatives if there’s a failure to identify crucial mutations. Thus, genetic data related to AMR phenotype, such as single nucleotide polymorphism (SNP), insertion/deletion, and copy number variation, gene recombination, horizontal gene transfer (HGT), and promoter mutations, are also an essential part of resistance prediction (13). For instance, Walker et al. (14) conducted WGS on 2099 *Mycobacterium tuberculosis* (MTB), characterizing 120 mutations as AMR determinants and successfully predicting 89.2% of the validation set phenotypes. Furthermore, some studies employ a combination of ARGs and SNPs as input features. For instance, Gordon et al. (7) predicted resistance to 12 antibiotics in *S. aureus* using 24 ARGs and their mutations. Similarly, Feuerriegel et al. (15) used 11 ARGs and a catalog of SNPs to define 92 strains as input features for resistance prediction based on WGS data from MTB, all of which resulted in effective prediction. Also, chromosomal variants have recently been reported to be useful for AMR phenotype identification. The *Pseudomonas aeruginosa* AMR prediction model constructed by Danielle et al. achieved more than 80% accuracy, which is a superior performance compared to previously reported tools, and its superior performance is mainly attributed to the inclusion of chromosomal AMR variants (16).

In addition to gene and mutation data, numerous k-mer-based models for predicting AMR phenotypes have been developed (13, 17–20). For instance, Wang et al. developed ML models for *S. aureus* based on the k-mer method, achieving prediction accuracies for AMR phenotypes over 80% (17). Similarly, Drouin et al. (18) developed 107 highly accurate models for predicting AMR phenotypes, covering 12 human pathogens and 56 antibiotics using k-mer data. These models not only demonstrated accuracy but also shed light on potential resistance mechanisms. Previous research suggests that k-mer features could be useful in predicting resistance and may aid in discovering new resistance mechanisms. However, it is often challenging to decipher the link between actual genetic factors and their representation through k-mer, which makes the underlying models difficult to interpret directly (21).

The performance of ML models based on single features still requires enhancement. In this study, we utilized 3,979 cases of *S. aureus* WGS data and incorporated three types of features—gene, SNP, and k-mer for model construction. We then assessed the performance of ML models for 10 common antibiotics in *S. aureus* using three algorithms: gradient boosting machine (GBM), generalized linear model (GLM), and random forests (RF). We also delineated the contribution of different features to the performance of ML models and worked toward discovering new molecular markers related to resistance determination in conserved regions to realize the improvement of molecular mechanisms related to *S. aureus* resistance.

## MATERIALS AND METHODS

### Strain WGS data and AST phenotype data acquisition

We collected the WGS raw FASTQ data and AST phenotypic information of the *S. aureus* strain from a public database. This included downloading the AST phenotype information of *S. aureus*, which indicates whether the strain is sensitive or resistant to specific antibiotics, along with the corresponding Sequence Read Archive (SRA) number from the Bacterial and Viral Bioinformatics Resource Center (BV-BRC) database. All included strains must meet the following criteria for the source of phenotype information: at least one experimentally measured AMR phenotype (MIC, disk diffusion, agar dilution, and Vitek2) is associated with the genome. Using the SRA numbers obtained from the BV-BRC database, we downloaded the corresponding raw genome sequencing data (FASTQ files) from the National Center for Biotechnology Information (NCBI) SRA database, selection of data with “GOOD” Genome Quality assessment. Ultimately, we acquired 3,979 genome sequencing data with AST phenotypic information. The numbers of resistant and sensitive strains are displayed in Table 1.

**TABLE 1:** Overview of the *S. aureus* AMR phenotype benchmark data set^*a*^

Antibiotic	Abbreviation	Resistant	Sensitive
Erythromycin	ERY	862	1,580
Ciprofloxacin	CIP	1,120	1,340
Clindamycin	CLI	404	619
Penicillin	PEN	1,096	176
Gentamicin	GEN	303	2,166
Methicillin	MRT	1,161	886
Tetracycline	TET	365	1,849
Trimethoprim/sulfamethoxazole	SXT	192	426
Fusidic acid	FUS	332	1847
Cefoxitin	FOX	898	251
Vancomycin	VAN	45	1,274
Rifampin	RIF	35	2,005
Oxacillin	OXA	65	427
Chloramphenicol	CHL	41	823

^*a*^ The number of resistant and susceptible strains of any antibiotic below 100 is not presented in the official results, and this part of the data is available in the supplemental material.

### Feature data acquisition

**Fig 1:**
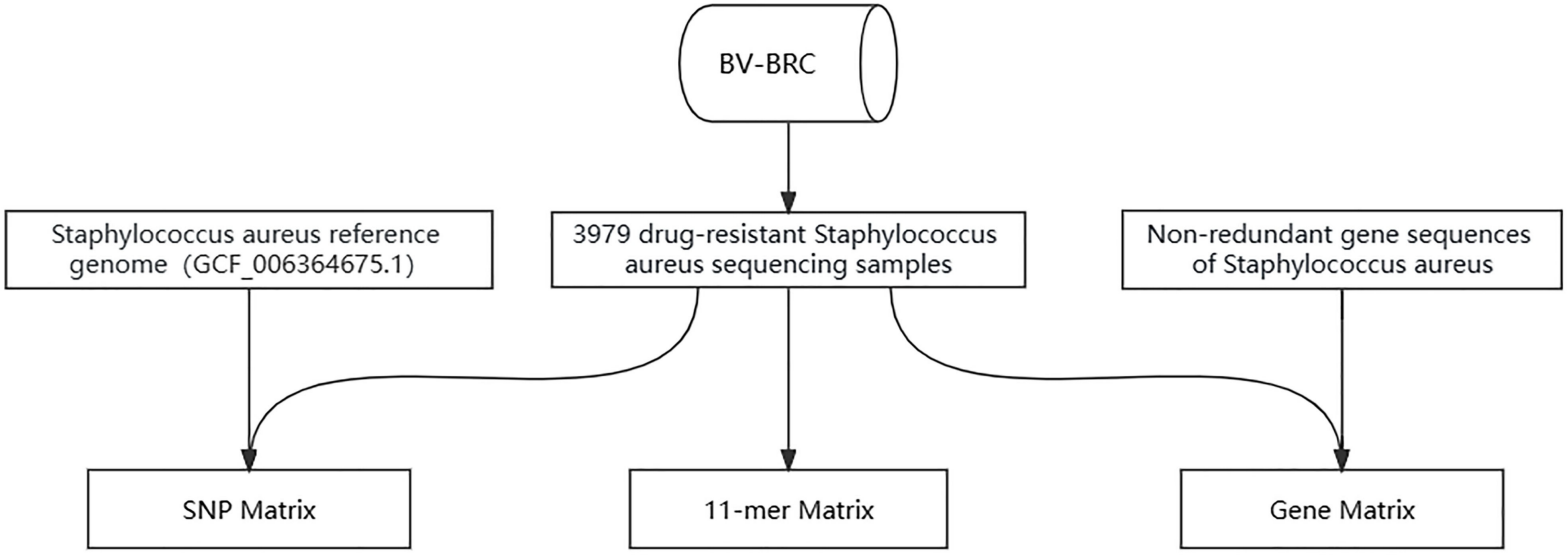
Data sources and processing. Based on the BV-BRC database, the data of 3979 *S*. *aureus* samples meeting the enrollment conditions were clarified, followed by the acquisition of three types of features. (i) Acquisition of SNP data: the detection of SNP deletion status, reference genome comparison (GCF_006364675.1), variant identification, and quality control. (ii) Gene data acquisition: obtain the coding sequences of the genome, delete redundant sequences, and retain the coding sequences with more than 90% coverage. (iii) Acquisition of k-mer data: set the length of the sequence as 11, obtain the k-mer sequences, quality control and de-weighting, and randomly select 100,000 11-mer sequences for the subsequent modeling analysis. The software and specific parameters involved are detailed in Materials and Methods.

Acquiring SNP data involved several steps. Initially, data from 3,979 samples were cleansed of low-quality sequences (samples with >60% missing SNP sites) using Fastp software (22). The high-quality data that passed this initial control were then compared to the *S. aureus* genome (GCF_006364675.1) using Burrows-Wheeler Aligner (BWA) software (23). For the final step, the GATK HaplotypeCaller software was used to identify variants and perform additional quality control (24). The filtering criteria for SNP locus included max-missing < 0.9, min-alleles = 2, max-alleles = 2, maf > 0.05, minQ > 30, minDP > 5, and min-meanDP > 3. In addition, samples were required to have less than 60% missing SNP loci (Fig. 1).

For gene data acquisition, the coding sequences of 1097 *S*. *aureus* genomes were retrieved from the NCBI, amounting to a total of 2,994,969 sequences. Cd-hit software was used to remove 2,976,433 redundant coding sequences (25), with a similarity and coverage threshold of more than 0.95 and 0.9, respectively. Sequencing data from 3,979 samples, along with the non-redundant genes, were then compared using BWA software. Only coding sequences with over 90% coverage were retained (Fig. 1).

For acquisition of k-mer data, we obtained sequencing data from 3,979 samples and processed them using Kmc software to derive k-mer sequences (26). We set the sequence length at 11 to maintain a balance between sequence specificity and computational efficiency. Next, we performed quality control on the k-mer sequences, removing duplicates and only keeping 11-mer sequences with detection rates between 1% and 99%. Lastly, we randomly selected 100,000 11-mer sequences to be used in our subsequent modeling analysis (Fig. 1).

### Model construction and cross-validation

First, we randomly divided all strains into training and validation sets, with 60% of the samples serving as the training set for model development. Within the training set, we utilized the χ test and the odds ratio (OR) method (*P* < 0.05 and OR >2) to identify drug resistance-related SNPs, genes, and k-mer data. We used the h2o.gbm, h2o.glm, and h2o.RF functions from the R package H2O to build models for the identified SNP, gene, and k-mer data, respectively. This allowed us to create ML models based on the three algorithms: GBM, GLM, and RF. We also integrated these models using the h2o.stackedEnsemble function to produce an AMR phenotype prediction model encompassing three types of features. We evaluated the effectiveness of all models using 10-fold cross-validation based on the sequencing data from the test set strains.

### Results and interpretation

The performance of the model was evaluated by comparing the AMR phenotype predictions from the validation set with the AST phenotype data. This evaluation was conducted using several criteria, including the ROC curve, area under the curve (AUC) value, sensitivity, specificity, F1-score, and accuracy. The data for all models are summarized, and the corresponding performance plots are drawn. In particular, the AUC value was derived from the area under the ROC curve and is a representation of the trade-off between sensitivity and 1-specificity. Sensitivity, the ratio of correctly predicted resistant outcomes to the actual number of resistant samples, and specificity, the ratio of correctly predicted susceptible outcomes to the actual number of susceptible samples, were also calculated. The F1-score, the harmonic mean of sensitivity and predictive value for a specific class (either susceptible or resistant), and accuracy, the proportion of samples correctly classified by the model, were also used in the evaluation. The performance metrics data for all models were compiled and visualized using box plots for easy comparison. These metrics provide a comprehensive overview of how accurately the classification model performs in the classification of AMR phenotypes.

## RESULTS

### Statistical analysis of strain origin and AST phenotypes

**Fig 2:**
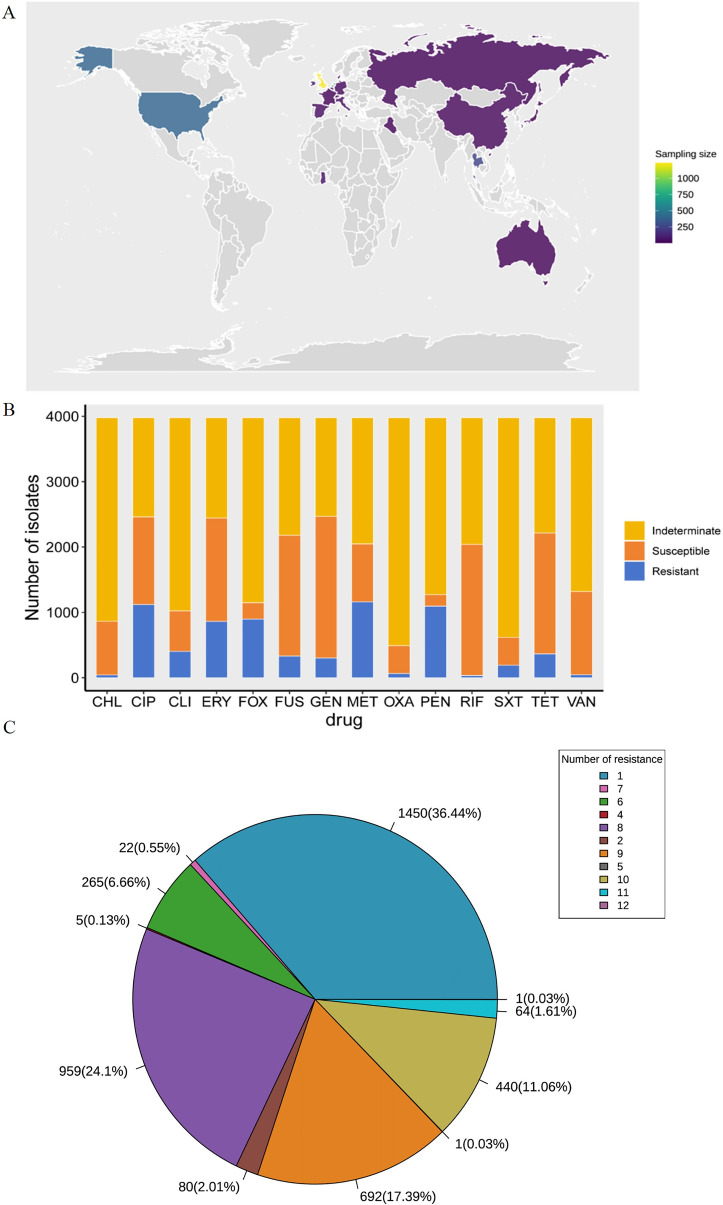
Geographical distribution and antibiotic resistance analysis of 3,979 *S*. *aureus* isolates used in this study. (**A**) Geographic sampling site distribution of strains. (**B**) Antimicrobial susceptibility profiles against the 14 commonly administered antibiotics. (**C**) Statistical chart of the number of strains simultaneously resistant to multiple antibiotics (Legends are presented by starting with resistance to one antibiotic and then sorting the legend counterclockwise based on the dark blue area of the pie chart).

The sequencing data for 3,979 cases of *S. aureus* primarily came from the United Kingdom, the United States, China, France, Germany, Russia, Australia, and other countries. These data spanned multiple regions, including Asia, Europe, and North America (Fig. 2A), while the multilocus sequence typing (MLST) typing data of 3,979 *S*. *aureus* cases showed some diversity in ST typing according to our download from the SRA database (Supplemental data), suggesting that the strains we included may have high genomic diversity. We then compiled the AST phenotypic data for all *S. aureus* isolates in the BV-BRC database for 14 commonly used antibiotics and plotted the corresponding resistance profiles (Fig. 2B). Approximately 61.55% of the *S. aureus* strains demonstrated resistance to more than three antibiotics, indicating a high percentage of multidrug-resistance strains, which are defined as those insensitive to three different antibiotics (Fig. 2C). We also considered that some antibiotics have a small number of resistant or sensitive strains (less than 100 cases) and a class imbalance of resistant and sensitive strains. This type of data could lead to overfitting in the model, potentially biasing model predictions. Therefore, we only assessed the performance of the model for ten antibiotics that met the inclusion criteria.

### ML models for predicting AMR

We conducted model construction utilizing WGS and AMR phenotype data from *S. aureus*. To assess the influence of different types of features on the model’s performance, we collected three types of data: gene, SNP, and k-mer. The SNP data were filtered, leaving 71,326 SNP loci and including 3,966 samples in the study. The gene data ultimately comprised 3,979 samples and included 14,878 genes. For the k-mer data, a total of 3,979 samples were retained, along with 100,000 11-mers.

**Fig 3:**
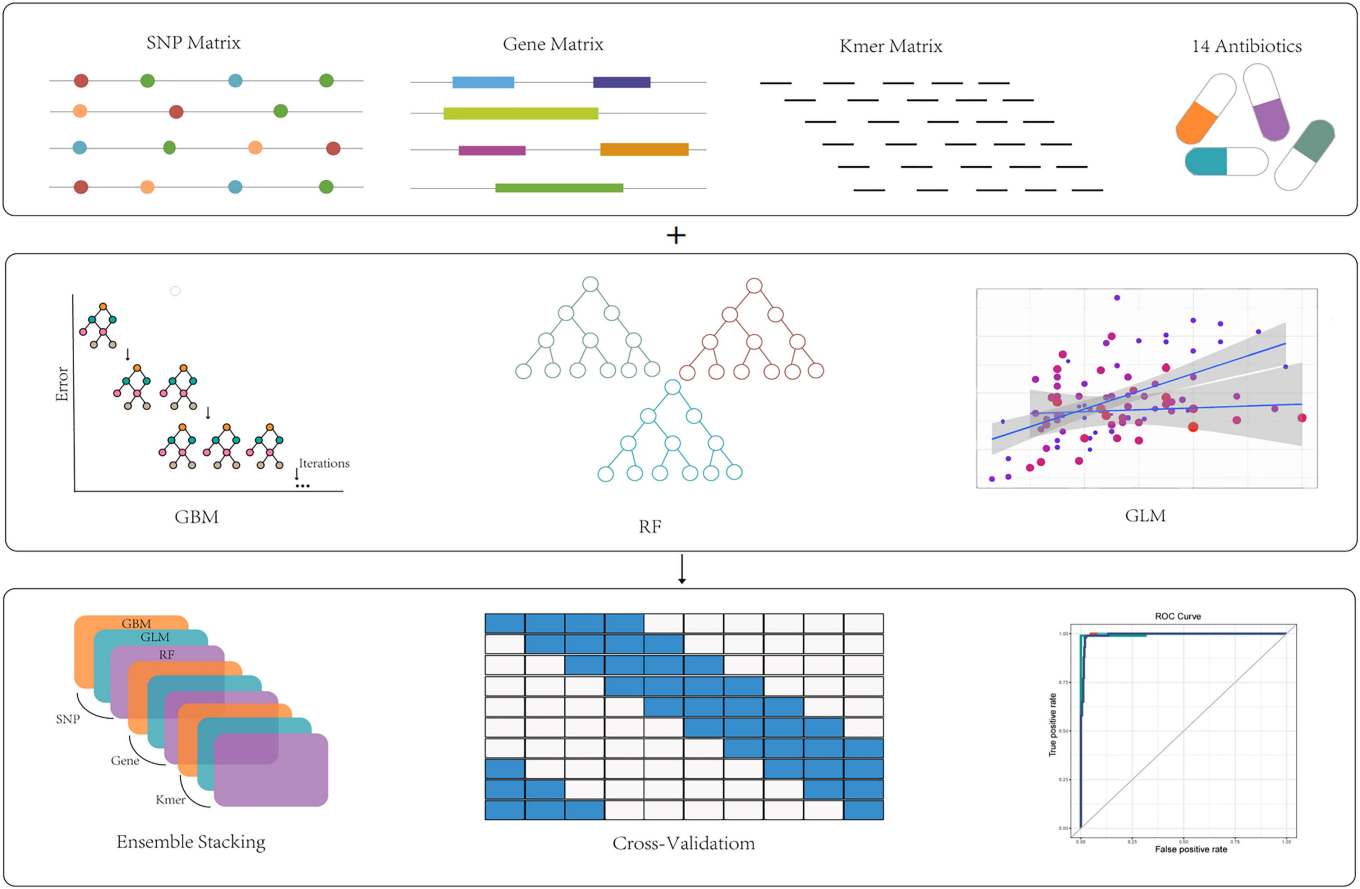
The process of ML classifier construction. Four models were constructed for each antibiotic, including three single-feature models constructed with genes, SNPs, and k-mer, and one multifeature model integrating genes, SNPs, and k-mer; three algorithms of GBM, RF, and GLM were used in the construction of all the models at the same time; the models were trained and validated using cross-validation. Finally, based on the validation set, we output the ROC curve graph and other evaluation indexes of each model performance.

We combined the selected genes, SNPs, and k-mer features to individually construct single-feature models and integrate multiple features based on three algorithms: GBM, GLM, and RF. For each antibiotic, we established four models, including three single-feature models and one integrated model. We selected 40% of the test set samples for cross-performance validation and evaluated the performance of both the individual and integrated models. We calculated the AUC values, sensitivity, specificity, accuracy, and F1 scores as overall performance indicators of classifiers trained on specific types of features. The workflow of this study is shown in Fig. 3.

### Model performance evaluation based on individual or integrated features

**Fig 4:**
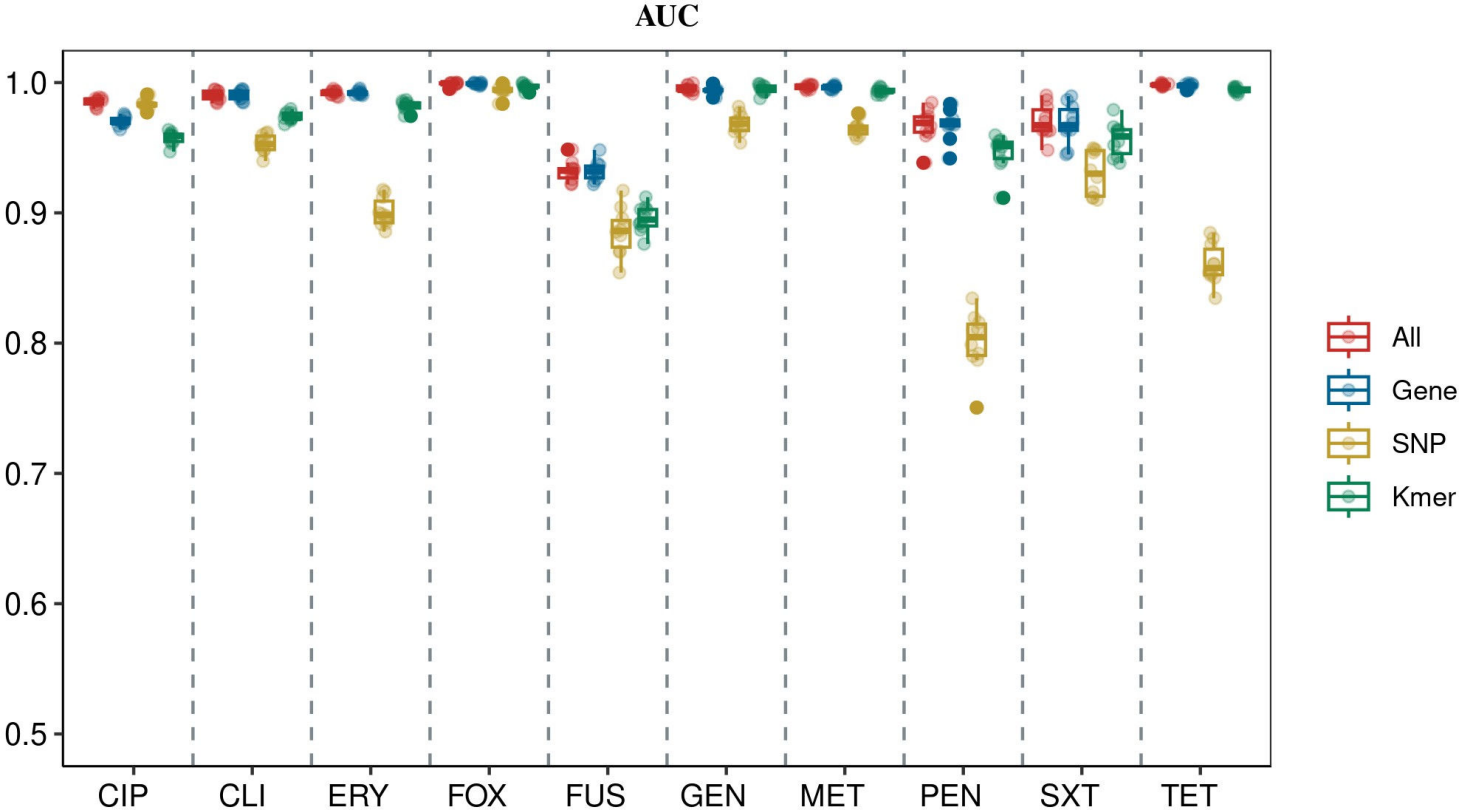
AUC performance plot of 40 ML models for 10 antibiotics in *S. aureus* (performance metrics were obtained based on validation set data).

**Fig 5:**
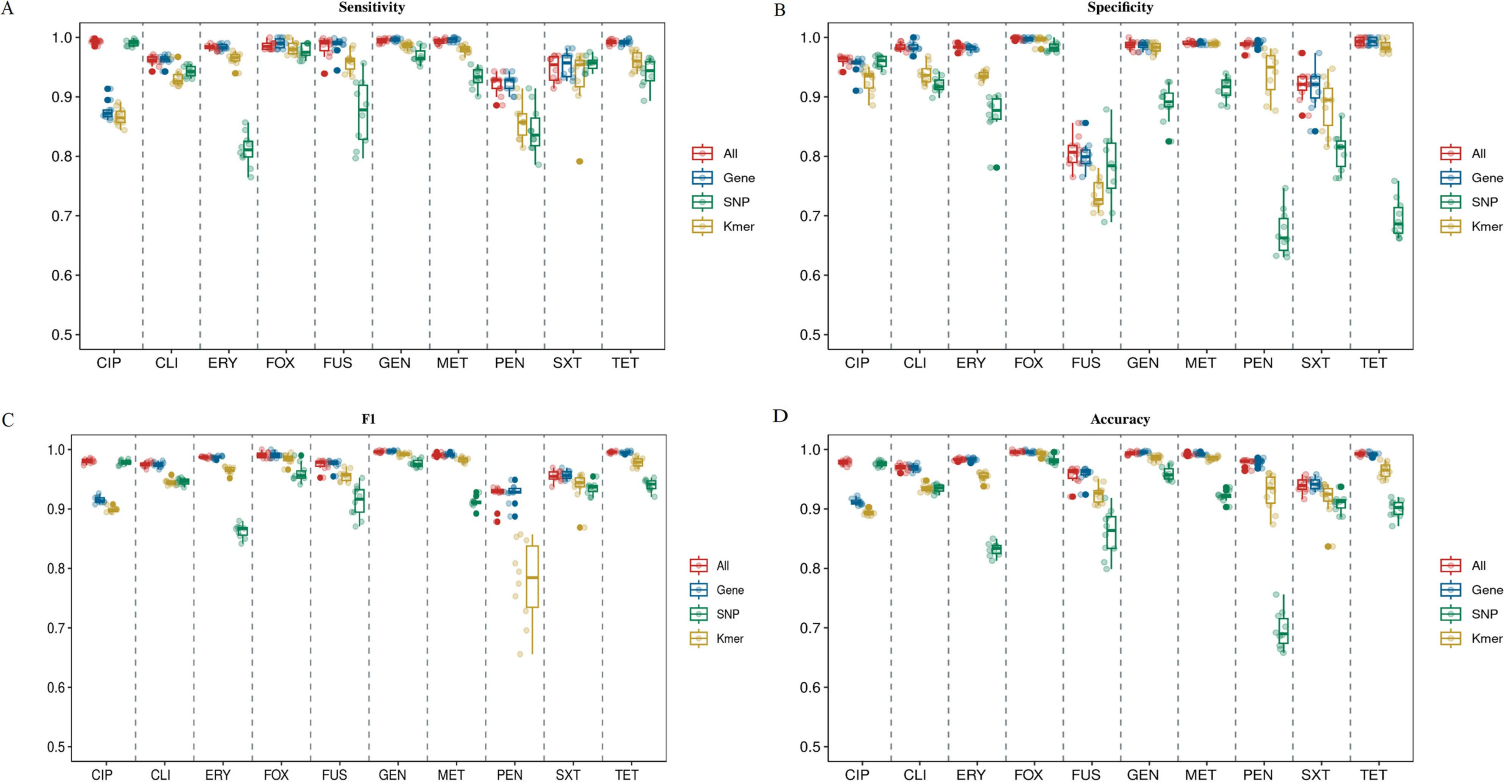
Plot of sensitivity (**A**), specificity (**B**), F1 value (**C**), and accuracy (D) of the 40-item ML models for 10 antibiotics in *S. aureus*.

The AUC values of the models for each category of the 10 antibiotics in the validation set are shown in Fig. 4 and Table 2; the AUC values of the 40 AMR prediction models for the 10 antibiotics ranged from 0.8345 to 0.9995, indicating that all the trained models were effective in making predictions as compared to the stochastic models. Meanwhile, we found that the AUC values of the 10 integration models ranged from 0.9313 to 0.9995, the AUC values of the 10 gene-based models ranged from 0.9311 to 0.9992, the AUC values of the k-mer as an input feature ranged from 0.9024 to 0.9969, and the AUC values of the SNP as an input feature ranged from 0.8345 to 0.9933. In addition to the AUC values, both the gene models and the integration models showed markedly better performance than the models constructed when SNPs and k-mer were used as features when analyzed in terms of accuracy, F1 values, sensitivity, and specificity (Fig. 5 and Table 2).

**TABLE 2:** Performance data of 40 ML models for 10 antibiotics in *S. aureus* validation set^*a*^

Antibiotic	Model	AUC	F1	Accuracy	Sensitivity	Specificity
Cefoxitin	Gene	0.99915	0.994975	0.997792	0.99	1
Cefoxitin	SNP	0.993258	0.951923	0.977925	0.99	0.974504
Cefoxitin	k-mer	0.996856	0.994975	0.997792	0.99	1
***Cefoxitin***	***All***	***0.999518***	***0.994975***	***0.997792***	***0.99***	***1***
Ciprofloxacin	Gene	0.968945	0.910891	0.907975	0.864662	0.959641
Ciprofloxacin	SNP	0.983854	0.974977	0.972393	0.988722	0.952915
Ciprofloxacin	k-mer	0.954801	0.896878	0.888548	0.890977	0.88565
***Ciprofloxacin***	***All***	***0.986379***	***0.979592***	***0.977505***	***0.992481***	***0.959641***
***Clindamycin***	***Gene***	***0.991333***	***0.979339***	***0.975062***	***0.971311***	***0.980892***
Clindamycin	SNP	0.953521	0.950413	0.94015	0.942623	0.936306
Clindamycin	k-mer	0.972747	0.94433	0.932668	0.938525	0.923567
Clindamycin	all	0.990864	0.979339	0.975062	0.971311	0.980892
***erythromycin***	***Gene***	***0.994365***	***0.988871***	***0.985522***	***0.988871***	***0.97929***
Erythromycin	SNP	0.88558	0.867227	0.836608	0.82035	0.866864
Erythromycin	k-mer	0.978784	0.959492	0.94726	0.960254	0.923077
Erythromycin	All	0.993217	0.988048	0.984488	0.985692	0.982249
Fusidic.acid	Gene	0.931105	0.977985	0.962069	0.993225	0.787879
Fusidic.acid	SNP	0.895782	0.894699	0.833333	0.834688	0.825758
Fusidic.acid	k-mer	0.902516	0.956347	0.925287	0.96477	0.704545
***Fusidic.acid***	***All***	***0.931295***	***0.977985***	***0.962069***	***0.993225***	***0.787879***
***Gentamicin***	***Gene***	***0.99442***	***0.998251***	***0.996933***	***0.997669***	***0.991667***
Gentamicin	SNP	0.965404	0.970484	0.948875	0.958042	0.883333
Gentamicin	k-mer	0.996843	0.991794	0.985685	0.986014	0.983333
Gentamicin	all	0.994221	0.998834	0.997955	0.998834	0.991667
***Methicillin***	***Gene***	***0.997161***	***0.991549***	***0.992647***	***0.997167***	***0.989201***
Methicillin	SNP	0.966712	0.912181	0.92402	0.912181	0.933045
Methicillin	k-mer	0.993374	0.985836	0.987745	0.985836	0.989201
Methicillin	All	0.996925	0.990127	0.991422	0.994334	0.989201
***Penicillin***	***Gene***	***0.967525***	***0.909091***	***0.974***	***0.928571***	***0.981395***
Penicillin	SNP	0.834468	0.453901	0.692	0.914286	0.655814
Penicillin	k-mer	0.950482	0.794521	0.94	0.828571	0.95814
Penicillin	All	0.966429	0.878378	0.964	0.928571	0.969767
Tetracycline	Gene	0.997571	0.991724	0.986301	0.983584	1
Tetracycline	SNP	0.875872	0.942857	0.90411	0.948016	0.682759
Tetracycline	k-mer	0.995627	0.976907	0.962329	0.954856	1
***Tetracycline***	***All***	***0.998113***	***0.992419***	***0.987443***	***0.984952***	***1***
***Trimethoprim-sulfamethoxazole***	***Gene***	***0.965289***	***0.963855***	***0.949791***	***0.981595***	***0.881579***
Trimethoprim-sulfamethoxazole	SNP	0.911568	0.918919	0.887029	0.93865	0.776316
Trimethoprim-sulfamethoxazole	k-mer	0.938247	0.936937	0.912134	0.957055	0.815789
Trimethoprim-sulfamethoxazole	All	0.96432	0.946708	0.92887	0.92638	0.934211

^*a*^ The bolded and italicized portions of the table show optimal model performance data for 10 antibiotics in *S. aureus* (AUC was used as the evaluation metric).

### AMR phenotype prediction performance of optimal models

For ML classifiers, the AUC value is a crucial metric for evaluating model performance, and it has been utilized several times in previous studies (10, 16, 27). Therefore, we selected optimal models for 10 antibiotics based on the AUC value criterion. Among the 10 optimal models, six were gene-based, while four utilized integrated data as features. The performance data of these optimal models for each antibiotic are presented in Table 2 with bold and italicized markers. The AUC values of the optimal models for six antibiotics—cefoxitin, tetracycline, methicillin, gentamicin, erythromycin, and clindamycin—exceeded 0.99. For nine antibiotics, except fusidic acid, the ML models exhibited excellent performance with AUC values surpassing 0.96. Fusidic acid, however, had a relatively lower AUC value of 0.9313 and poorer model specificity among the 10 AMR phenotypic prediction models. We speculate that this may be attributed to the class imbalance between resistant and sensitive strains.

### Contribution of different types of biomarkers to AMR phenotype prediction

Upon analyzing the performance data of the 40 mL models, we confirmed that gene features hold greater value in predicting *S. aureus* AMR phenotypes. Both gene-based models and integration models exhibited superior performance. Specifically, for methicillin, gentamicin, erythromycin, clindamycin, penicillin, and trimethoprim-sulfamethoxazole, the gene model achieved the highest AUC values, underscoring the significance of gene features in AMR prediction. Conversely, the inclusion of additional k-mer and SNP information resulted in decreased model performance. We speculate that this might be attributed to the excessive number of features, leading to decreased model sparsity and, subsequently, impacting model performance and reducing runtime speed. Therefore, feature selection is deemed crucial for different antibiotics. However, for antibiotics such as cefoxitin, ciprofloxacin, tetracycline, and fusidic acid, incorporating SNP and k-mer information could enhance the model’s AUC values to some extent. Furthermore, based on sensitivity, specificity, F1 score, and accuracy metrics, both the gene model and the integrated model demonstrated superior performance. These findings indicate that gene characterization is an indispensable tool for predicting resistance in *S. aureus*. Nevertheless, for certain antibiotics, augmenting the model with SNP and k-mer information can further improve prediction accuracy.

### Feature importance assessment

**Fig 6:**
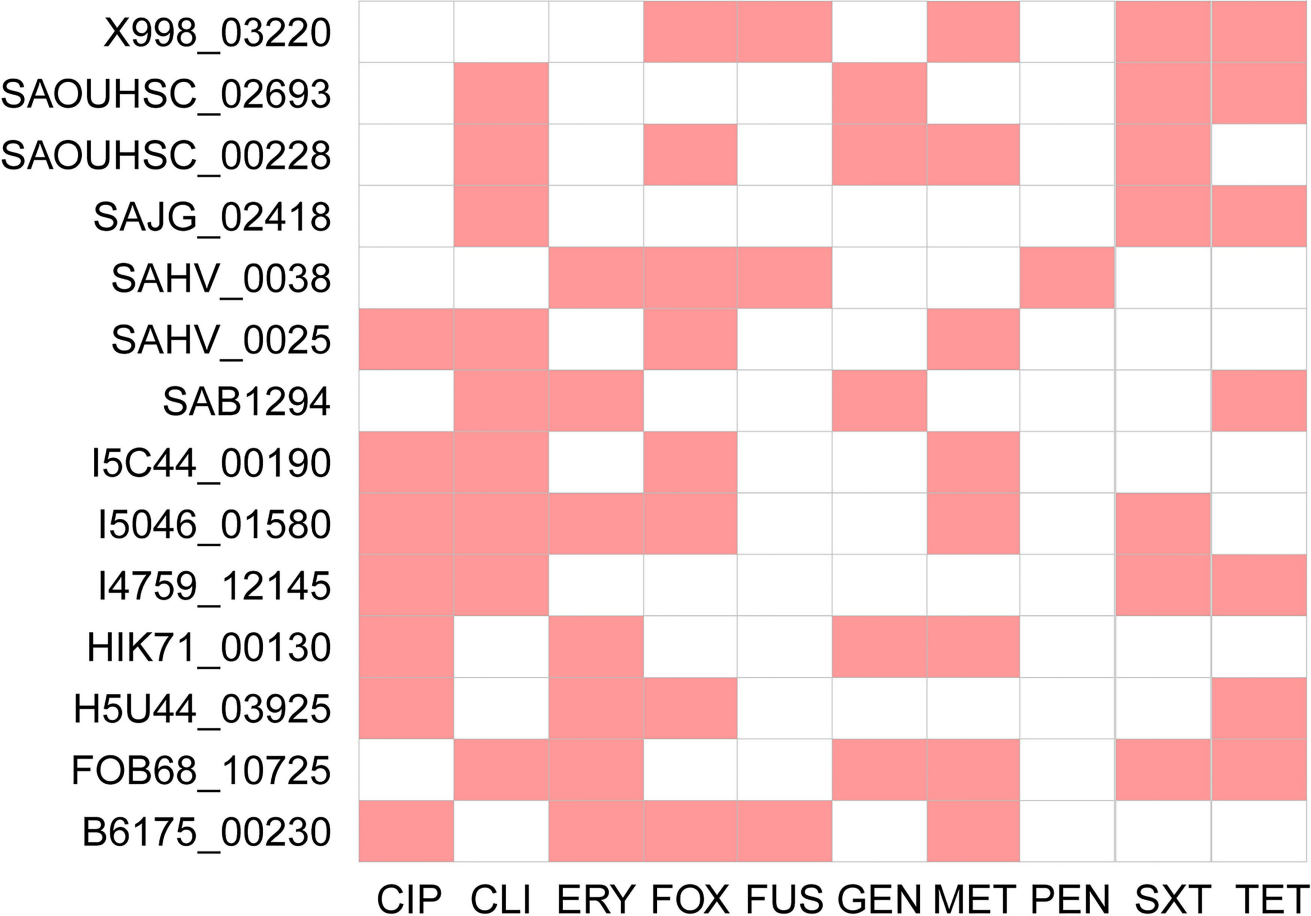
Genomic map for discriminating AMR phenotypes across 10 antibiotics in *S. aureus* (red markers represent a higher contribution of the gene to the differentiation of resistance/sensitivity to this antibiotic, while blanks indicate a lower contribution).

When developing a model, it is crucial to identify genes that play a role in distinguishing between sensitive and resistant strains. Upon extracting the significant genes contributing to the models, as indicated in Fig. 6, we discovered that a total of 14 genes exhibited varying contributions across the prediction models for 10 antibiotics and the ID and location information of each gene in NCBI is detailed in Table S3. Specifically, genes FOB68_10725 and I5046_01580 were found to contribute to the prediction models for six antibiotics. Additionally, gene X998_03220 not only contributed to differentiating between sensitive and resistant strains for cefoxitin, fusidic acid, methicillin, tetracycline, and trimethoprim-sulfamethoxazole but also played a role in predicting rifampin and oxacillin resistance, which were not initially described in the results (Fig. S1). The X998_03220 gene encodes heat-stable nucleoid-structuring (H-NS), known to regulate the expression of AMR-related genes in *Acinetobacter baumannii* (28). Despite being less studied in *S. aureus*, this gene could serve as a potential candidate for further exploration of its role in resistance mechanisms.

## DISCUSSION

Previous ML models focused on predicting *S. aureus* AMR phenotypes typically used single or double feature combinations (16, 17, 27). For instance, Wang et al. built a model based on the k-mer extraction information from 466 *S*. *aureus* genomes, achieving AUC values ranging from 0.82 to 0.96 for 10 different antibiotics (16). Hyun et al. (27) not only successfully predicted AMR phenotypes of *S. aureus* using pan-genomic genetic information but also identified the genetic traits that drive AMR. Yang and Wu (10) developed models based on gene presence/absence of pan-genomes from four bacterial species, including *S. aureus*, with AUC values around 0.95, thereby expanding the library of AMR-related genes. However, due to the complex mechanisms of AMR, such as gene overexpression or duplication, point mutations, and HGT (29), some phenotype predictions may not be accurate if the characterization is not perfect. Hence, there is still potential for improving the accuracy of ML models that rely on single features in AMR phenotype prediction. In our study, we used three features both individually and collectively for model construction and then compared their performance to build the best model. Among the best models for 10 antibiotics, the AUC value for six was greater than 0.99, and for nine antibiotics (excluding fusidic acid), it was greater than 0.96, indicating excellent performance. Our findings suggest that, without any prior knowledge of the molecular mechanism of AMR, ML methods that use gene, SNP, and k-mer features can provide highly accurate AMR phenotype predictions for *S. aureus*.

Comparing the model performance with different feature types, we found that the gene model (0.9311–0.9992) and the integration model (0.9313–0.9995) significantly outperformed the SNP model (0.8345–0.9933) and the k-mer model (0.9024–0.9969); this was evident across 10 antibiotics and was assessed using performance indicators such as AUC. In this study, we found that the inclusion of SNP and k-mer information could enhance the performance of the model for cefoxitin, tetracycline, ciprofloxacin, and fusidic acid when compared to the gene model. This suggests that for certain antibiotics, integrating different types of biomarkers can improve model performance to varying extents. It also reflects that different biomarkers play unique roles in AMR phenotype prediction, with each having a different level of importance. This contribution of various biomarkers in AMR phenotype prediction has also been observed in *P. aeruginosa*. Khaledi et al. demonstrated that adding gene expression information dramatically improved prediction sensitivity for ceftazidime. This confirms that expanding the genetic signature is crucial for enhancing the accuracy of ML classifiers (30). Contrarily, the ML classifiers showed better performance in the case of methicillin, gentamicin, erythromycin, clindamycin, penicillin, and trimethoprim-sulfamethoxazole when individual genes were used for modeling. This could be due to the combined use of the three features, which to some extent, reduced the model’s sparsity, and the combined effect of different AMR mechanisms was not effectively captured. We hypothesize that the influence of different biomarker classes on the model’s performance is largely dependent on the specific antibiotics.

Furthermore, this study utilized all genes as input features rather than known ARGs and observed that the gene model demonstrated the best performance among the six antibiotic models, including methicillin. This finding reflects the significant contribution of gene features to the ML model for multiple antibiotics of *S. aureus*. Additionally, our study identified a substantial number of non-AMR functional genes among the features that made strong contributions to the ML model. We speculate that these genes may be associated with the discovery of conserved regions of non-ARGs. Moreover, these genes were found to overlap in the ML models of 10 antibiotics, with FOB68_10725 and I5046_01580 contributing to the ML models of 6 antibiotics, and X998_03220 contributing to the ML model performance of cefoxitin, fusidic acid, methicillin, tetracycline, and trimethoprim-sulfamethoxazole, in addition to contributing to the distinction of the AMR phenotype in the ML models of rifampin and oxacillin, although the latter was not explicitly described in the results. These findings expand the pool of potential resistance genes, to some extent contributing to the elucidation of the AMR mechanism in *S. aureus*, and validate the potential overlap of AMR-related features across different antibiotics. The X998_03220 gene was demonstrated to encode H-NS (28), and the role of H-NS proteins in AMR was established in extensive studies, and in *P. aeruginosa*, the H-NS-like proteins MvaT and MvaU coordinately acted as global repressors to promote *P. aeruginosa* growth through coordinated silencing of intragenic transcription (31). Additionally, H-NS was also observed to inhibit biofilm formation and c-di-GMP synthesis in *Vibrio parahaemolyticus* (32). In *Vibrio cholerae* and *Enterobacteriaceae*, N-HS is suggested to regulate gene expression by inhibiting transcription (33–36). In *A. baumannii*, H-NS also plays a role in regulating the expression of AMR-related genes (28). Our study identifies novel non-known ARGs driving the prediction of *S. aureus* AMR phenotypes, which could contribute to the clarification of *S. aureus* AMR mechanisms and those related to acquired drug resistance.

For vancomycin, rifampin, oxacillin, and chloramphenicol, despite their clinical importance, we did not present the relevant data in our results because the number of resistant strains was below 100, and there was a severe category imbalance with the number of sensitive strains (Table S1). Due to the tendency of most standard algorithms to favor phenotypes of larger classes while neglecting phenotypes with smaller sample sizes, prediction accuracy is adversely affected (37–39). In the supplementary material, the optimal model AUC values for vancomycin, chloramphenicol, and oxacillin all exceeded 0.98. However, we noted that the rifampicin model achieved a subpar performance with an optimal AUC value of 0.817, which was lower compared to the other 13 antibiotic models. And the inclusion of the resistant *S. aureus* strains in the present study was only 35 strains, while sensitive strains as high as 2,005 strains. The reason for the relatively poor model performance may be related to the extreme imbalance between resistant and sensitive strains. Furthermore, among the rifampicin ML models, the SNP model demonstrated the highest AUC value of 0.817, surpassing both the gene model (0.759) and the k-mer model (0.682), distinguishing it from the other 13 antibiotics. Rifampicin exerts its antimicrobial effect mainly by inhibiting the transcription process through binding to rpoB (40). And the frequent mutation of rpoB in the rifampicin resistance determining region resulted in the reduction of rifampicin binding efficiency, which in turn led to the enhancement of rifampicin resistance (41), suggesting that the mutation information plays a remarkable role in the prediction of AMR phenotypes of rifampicin. Our research indicates that the key feature types of AMR phenotype prediction models may vary for different classes of antibiotics, emphasizing the importance of selecting optimal molecular markers for targeted ML model construction to achieve the best performance.

Although our models demonstrate relatively superior performance, with some antibiotic ML models achieving prediction accuracies nearing 100%, there are notable limitations in our study. Specifically, certain antibiotics like vancomycin hold clinical significance; however, we face a significant imbalance in the number of resistant and susceptible strains available to us. This imbalance may contribute to model overfitting and diminish prediction accuracy. Future studies will focus on optimizing ML model construction for such antibiotics with category imbalances. Additionally, for guiding clinical practices, incorporating MIC indicators into binary classification problems for predicting drug resistance or sensitivity holds promise. Unfortunately, most public databases lack MIC information. Moving forward, we aim to gather clinical data to enhance model performance and address this limitation.

In conclusion, our study demonstrates that comprehensive genetic characterization, leveraging a combination of different biomarkers, is crucial for enhancing model performance, although its applicability may vary across different antibiotic types. Furthermore, the utilization of pan-genomic-based characterization has uncovered novel potential molecular markers associated with AMR, thereby advancing our understanding of the molecular mechanisms underlying *S. aureus* AMR. Additionally, our research has identified several genes that play a role in multiple antibiotic ML models, albeit to a limited extent. Future investigations should aim to broaden the scope of captured molecular markers associated with *S. aureus* drug resistance. As we gain further insights into the AMR mechanism, the expedited implementation of early and targeted antimicrobial therapy for *S. aureus* infections will advance precision medicine and contribute to reducing healthcare costs.

## Data Availability

The data sets used in this study are open access. *S. aureus* AST phenotypes, and corresponding SRA numbers were obtained from the BV-BRC database (https://www.bv-brc.org/); the corresponding raw genome sequencing data (FASTQ files) were downloaded from the NCBI database (https://www.ncbi.nlm.nih.gov/sra/). The data sets generated during and/or analyzed during the current study are available from the corresponding author on reasonable request.

## SUPPLEMENTAL MATERIAL

Supplemental material spectrum.00065-25-s0001.pdf Tables S1 to S3; Fig. S1.

Supplemental data spectrum.00065-25-s0002.xlsx SRA data.

ASM does not own the copyrights to Supplemental Material that may be linked to, or accessed through, an article. The authors have granted ASM a non-exclusive, world-wide license to publish the Supplemental Material files. Please contact the corresponding author directly for reuse.
