# Supplementary material for: Prediction of antimicrobial resistance in Staphylococcus aureus with a machine learning classifier based on WGS data
Source: Microbiol Spectr. 2025 Aug 5;13(9):e00065-25. doi: 10.1128/spectrum.00065-25 (PMC12403771; doi:10.1128/spectrum.00065-25)
Supplement: Supplemental material — Tables S1 to S3; Fig. S1. [file spectrum.00065-25-s0001.pdf]

Supplementary material

Supplementary Table 1. ML model performance data for four antibiotics including vancomycin in *S. aureus* based on the integration algorithm (data from validation set).

Antibiotic	Model	AUC	F1	Accuracy	Sensitivity	Specificity
rifampin	Gene	0.759870	0.919431	0.852357	0.856242	0.615384
rifampin	SNP	0.817150	0.947159	0.900744	0.904161	0.692307
rifampin	k-mer	0.682073	0.961663	0.926799	0.933165	0.538461
rifampin	All	0.798962	0.968952	0.940446	0.944514	0.692307
oxacillin	Gene	0.982142	0.966360	0.941489	0.940476	0.95
oxacillin	SNP	0.977678	0.959752	0.930851	0.922619	1
oxacillin	k-mer	0.972916	0.972809	0.952127	0.958333	0.9
oxacillin	all	0.983333	0.969512	0.946808	0.946428	0.95
vancomycin	Gene	1	1	1	1	1
vancomycin	SNP	1	1	1	1	1
vancomycin	k-mer	1	1	1	1	1
vancomycin	all	1	1	1	1	1
chloramphenicol	Gene	0.999809	0.998473	0.997093	0.996951	1
chloramphenicol	SNP	0.974275	0.979907	0.962209	0.966463	0.875
chloramphenicol	k-mer	0.993330	0.963665	0.933139	0.929878	1
chloramphenicol	all	0.999238	0.993865	0.988372	0.987804	1

Supplementary Table 2. ML model performance data for 14 antibiotics in S. aureus based on separate algorithms and single features (data from validation set).

Antibiotic	Model	AUC	F1	Accuracy	Sensitivity	Specificity
cefoxitin	Gene_GBM	0.9993484	0.994975	0.997792	0.99	1
cefoxitin	Gene_GLM	0.9976771	0.99	0.995585	0.99	0.997167
cefoxitin	Gene_RF	0.9991643	0.994975	0.997792	0.99	1
cefoxitin	SNP_GBM	0.9953824	0.961165	0.98234	0.99	0.98017
cefoxitin	SNP_GLM	0.9371388	0.716981	0.834437	0.95	0.8017
cefoxitin	SNP_RF	0.9904674	0.915094	0.960265	0.97	0.957507
cefoxitin	kmer_GBM	0.9965156	0.985075	0.993377	0.99	0.994334
cefoxitin	kmer_GLM	0.9937394	0.985075	0.993377	0.99	0.994334
cefoxitin	kmer_RF	0.9957507	0.99	0.995585	0.99	0.997167
chloramphenicol	Gene_GBM	0.9980945	0.98452	0.97093	0.969512	1
chloramphenicol	Gene_GLM	0.9988567	0.995406	0.991279	0.990854	1
chloramphenicol	Gene_RF	0.9992378	0.998473	0.997093	0.996951	1
chloramphenicol	SNP_GBM	0.9448361	0.976744	0.956395	0.960366	0.875
chloramphenicol	SNP_GLM	0.9636052	0.878632	0.793605	0.783537	1
chloramphenicol	SNP_RF	0.9860899	0.984568	0.97093	0.972561	0.9375
chloramphenicol	kmer_GBM	0.9807546	0.958861	0.924419	0.92378	0.9375
chloramphenicol	kmer_GLM	0.9645579	0.891892	0.813953	0.804878	1
chloramphenicol	kmer_RF	0.9922828	0.975078	0.953488	0.954268	0.9375
ciprofloxacin	Gene_GBM	0.9684118	0.908734	0.904908	0.870301	0.946188
ciprofloxacin	Gene_GLM	0.9571694	0.896347	0.892638	0.853383	0.939462

ciprofloxacin	Gene_RF	0.9687806	0.914563	0.91002	0.885338	0.939462
ciprofloxacin	SNP_GBM	0.9845557	0.975926	0.973415	0.990602	0.952915
ciprofloxacin	SNP_GLM	0.8933207	0.861568	0.853783	0.836466	0.874439
ciprofloxacin	SNP_RF	0.9691409	0.930403	0.92229	0.954887	0.883408
ciprofloxacin	kmer_GBM	0.9522531	0.895038	0.887526	0.881579	0.894619
ciprofloxacin	kmer_GLM	0.937795	0.86965	0.862986	0.840226	0.890135
ciprofloxacin	kmer_RF	0.9519897	0.888674	0.882413	0.862782	0.90583
clindamycin	Gene_GBM	0.98948	0.973085	0.967581	0.963115	0.974522
clindamycin	Gene_GLM	0.9908374	0.977131	0.972569	0.963115	0.987261
clindamycin	Gene_RF	0.9899629	0.966527	0.9601	0.946721	0.980892
clindamycin	SNP_GBM	0.9568367	0.944559	0.932668	0.942623	0.917197
clindamycin	SNP_GLM	0.942897	0.931174	0.915212	0.942623	0.872611
clindamycin	SNP_RF	0.9498799	0.92437	0.910224	0.901639	0.923567
clindamycin	kmer_GBM	0.9758275	0.943867	0.932668	0.930328	0.936306
clindamycin	kmer_GLM	0.9539261	0.921811	0.905237	0.918033	0.88535
clindamycin	kmer_RF	0.9429884	0.925926	0.910224	0.922131	0.89172
erythromycin	Gene_GBM	0.9922508	0.989658	0.986556	0.988871	0.982249
erythromycin	Gene_GLM	0.9896661	0.986454	0.98242	0.984102	0.97929
erythromycin	Gene_RF	0.9936219	0.979167	0.973113	0.971383	0.976331
erythromycin	SNP_GBM	0.8797213	0.863826	0.831437	0.82194	0.849112
erythromycin	SNP_GLM	0.8153475	0.82899	0.782834	0.809221	0.733728
erythromycin	SNP_RF	0.881257	0.858347	0.826267	0.809221	0.857988
erythromycin	kmer_GBM	0.9764701	0.957361	0.945191	0.945946	0.943787
erythromycin	kmer_GLM	0.9355251	0.91879	0.894519	0.917329	0.852071
erythromycin	kmer_RF	0.9388058	0.930631	0.907963	0.949126	0.831361
fusidic.acid	Gene_GBM	0.9241706	0.975251	0.957471	0.987805	0.787879
fusidic.acid	Gene_GLM	0.8900335	0.979375	0.964368	0.99729	0.780303
fusidic.acid	Gene_RF	0.9298473	0.978015	0.962069	0.99458	0.780303
fusidic.acid	SNP_GBM	0.8907161	0.907791	0.851724	0.860434	0.80303
fusidic.acid	SNP_GLM	0.7762483	0.922139	0.867816	0.922764	0.560606
fusidic.acid	SNP_RF	0.8814004	0.915374	0.862069	0.879404	0.765152
fusidic.acid	kmer_GBM	0.8911575	0.932965	0.888506	0.914634	0.742424
fusidic.acid	kmer_GLM	0.8914039	0.952381	0.918391	0.96206	0.674242
fusidic.acid	kmer_RF	0.8779615	0.959306	0.929885	0.974255	0.681818
gentamicin	Gene_GBM	0.9948378	0.998253	0.996933	0.998834	0.983333
gentamicin	Gene_GLM	0.9955177	0.994734	0.990798	0.990676	0.991667
gentamicin	Gene_RF	0.9949349	0.998834	0.997955	0.998834	0.991667
gentamicin	SNP_GBM	0.9673805	0.975925	0.958078	0.968531	0.883333
gentamicin	SNP_GLM	0.9034771	0.943577	0.903885	0.916084	0.816667
gentamicin	SNP_RF	0.9562549	0.978426	0.962168	0.977855	0.85
gentamicin	kmer_GBM	0.9976787	0.992393	0.986708	0.988345	0.975
gentamicin	kmer_GLM	0.9916035	0.982311	0.969325	0.970862	0.958333
gentamicin	kmer_RF	0.975777	0.981243	0.96728	0.975524	0.908333
methicillin	Gene_GBM	0.9963656	0.991549	0.992647	0.997167	0.989201
methicillin	Gene_GLM	0.9930892	0.991549	0.992647	0.997167	0.989201
methicillin	Gene_RF	0.9966073	0.990127	0.991422	0.994334	0.989201
methicillin	SNP_GBM	0.9635154	0.912429	0.92402	0.915014	0.930886
methicillin	SNP_GLM	0.8858473	0.818182	0.848039	0.790368	0.892009

methicillin	SNP_RF	0.9586482	0.903319	0.917892	0.886686	0.941685
methicillin	kmer_GBM	0.9945331	0.98727	0.988971	0.988669	0.989201
methicillin	kmer_GLM	0.9834403	0.965812	0.970588	0.96034	0.978402
methicillin	kmer_RF	0.9916238	0.970043	0.974265	0.963173	0.982721
oxacillin	Gene_GBM	0.9866071	0.96319	0.93617	0.934524	0.95
oxacillin	Gene_GLM	0.9901786	0.956522	0.925532	0.916667	1
oxacillin	Gene_RF	0.98125	0.96	0.930851	0.928571	0.95
oxacillin	SNP_GBM	0.9794643	0.956522	0.925532	0.916667	1
oxacillin	SNP_GLM	0.9849702	0.978979	0.962766	0.970238	0.9
oxacillin	SNP_RF	0.9705357	0.936709	0.893617	0.880952	1
oxacillin	kmer_GBM	0.9741071	0.975904	0.957447	0.964286	0.9
oxacillin	kmer_GLM	0.9610119	0.975904	0.957447	0.964286	0.9
oxacillin	kmer_RF	0.975	0.97281	0.952128	0.958333	0.9
penicillin	Gene_GBM	0.9654153	0.901408	0.972	0.914286	0.981395
penicillin	Gene_GLM	0.9645349	0.90411	0.972	0.942857	0.976744
penicillin	Gene_RF	0.9656146	0.872483	0.962	0.928571	0.967442
penicillin	SNP_GBM	0.8230399	0.471616	0.758	0.771429	0.755814
penicillin	SNP_GLM	0.8001661	0.423841	0.652	0.914286	0.609302
penicillin	SNP_RF	0.8243522	0.453237	0.696	0.9	0.662791
penicillin	kmer_GBM	0.9319934	0.743902	0.916	0.871429	0.923256
penicillin	kmer_GLM	0.9273588	0.764331	0.926	0.857143	0.937209
penicillin	kmer_RF	0.9383223	0.638298	0.864	0.857143	0.865116
rifampin	Gene_GBM	0.7543894	0.863863	0.763027	0.764187	0.692308
rifampin	Gene_GLM	0.797701	0.902826	0.825062	0.825977	0.769231
rifampin	Gene_RF	0.740518	0.938911	0.885856	0.891551	0.538462
rifampin	SNP_GBM	0.7747114	0.92814	0.867246	0.871375	0.615385
rifampin	SNP_GLM	0.8130275	0.897507	0.816377	0.81715	0.769231
rifampin	SNP_RF	0.8089048	0.920785	0.854839	0.857503	0.692308
rifampin	kmer_GBM	0.7198079	0.958279	0.920596	0.92686	0.538462
rifampin	kmer_GLM	0.7230575	0.932441	0.87469	0.878941	0.615385
rifampin	kmer_RF	0.6820739	0.961663	0.926799	0.933165	0.538462
tetracycline	Gene_GBM	0.9964857	0.991724	0.986301	0.983584	1
tetracycline	Gene_GLM	0.9961272	0.991724	0.986301	0.983584	1
tetracycline	Gene_RF	0.9970376	0.988935	0.981735	0.978112	1
tetracycline	SNP_GBM	0.8653333	0.949266	0.913242	0.97264	0.613793
tetracycline	SNP_GLM	0.8206472	0.927989	0.878995	0.934337	0.6
tetracycline	SNP_RF	0.8727676	0.934708	0.891553	0.930233	0.696552
tetracycline	kmer_GBM	0.9950469	0.971871	0.954338	0.94528	1
tetracycline	kmer_GLM	0.9114722	0.950103	0.916667	0.950752	0.744828
tetracycline	kmer_RF	0.9478183	0.929134	0.886986	0.887825	0.882759
trimethoprim_sulfamethoxazole	Gene_GBM	0.9750565	0.953271	0.937238	0.93865	0.934211
trimethoprim_sulfamethoxazole	Gene_GLM	0.969083	0.946708	0.92887	0.92638	0.934211
trimethoprim_sulfamethoxazole	Gene_RF	0.9468034	0.952663	0.933054	0.98773	0.815789
trimethoprim_sulfamethoxazole	SNP_GBM	0.9070471	0.925373	0.895397	0.95092	0.776316

trimethoprim_sulfamethoxazole	SNP_GLM	0.8897724	0.919403	0.887029	0.944785	0.763158
trimethoprim_sulfamethoxazole	SNP_RF	0.9018405	0.916168	0.882845	0.93865	0.763158
trimethoprim_sulfamethoxazole	kmer_GBM	0.9445835	0.943284	0.920502	0.969325	0.815789
trimethoprim_sulfamethoxazole	kmer_GLM	0.9170972	0.841379	0.807531	0.748466	0.934211
trimethoprim_sulfamethoxazole	kmer_RF	0.9226267	0.930514	0.903766	0.944785	0.815789
vancomycin	Gene_GBM	0.9722222	0.999009	0.998084	1	0.944444
vancomycin	Gene_GLM	1	1	1	1	1
vancomycin	Gene_RF	1	1	1	1	1
vancomycin	SNP_GBM	1	1	1	1	1
vancomycin	SNP_GLM	1	1	1	1	1
vancomycin	SNP_RF	1	1	1	1	1
vancomycin	kmer_GBM	0.9990079	0.999007	0.998084	0.998016	1
vancomycin	kmer_GLM	0.9993386	0.999007	0.998084	0.998016	1
vancomycin	kmer_RF	1	1	1	1	1

Supplementary Table 3. Table of ID names and locations in the NCBI database corresponding to the genes identified in this study.

Gene name	Gene ID（NCBI）	Gene location
X998_0322	/	CP007539.3: 2347065..2347454
SAOUHSC_02693	3919712	NC_007795.1: 2477863..2477964
SAOUHSC_00228	3920303	NC_007795.1（248727..250451
SAJG_02418	/	NZ_ACKG01000021.1 :9871-10011
SAHV_0038	5559278	NC_009782.1: 43678..44421
SAHV_0025	5559317	NC_009782.1: 34494..35750
SAB1294	3793782	NC_007622.1: 1416839..1417003
I5C44_00190	mecA| Q5HK31	CP065868.1: 43045-45005）
I5046_01580	3921677	1 CP065355.1: 356586-356771 NC_007795.1: 366986-367195
I4759_12145	Q2FXH8	NZ_JADSDQ010000008.1: 69281-69625
HIK71_00130	IS6 family transposase P14506	CP051960.1: 20433-21119
H5U44_03925	mecA| Q5HK31	CP060141.1: 801834-803844
FOB68_10725	3919876	CP044106.1: 2074429-2075969 NC_007795.1: 184615-186390
B6175_00230	mecA| Q5HK31	CP020553.1: 46816-48848

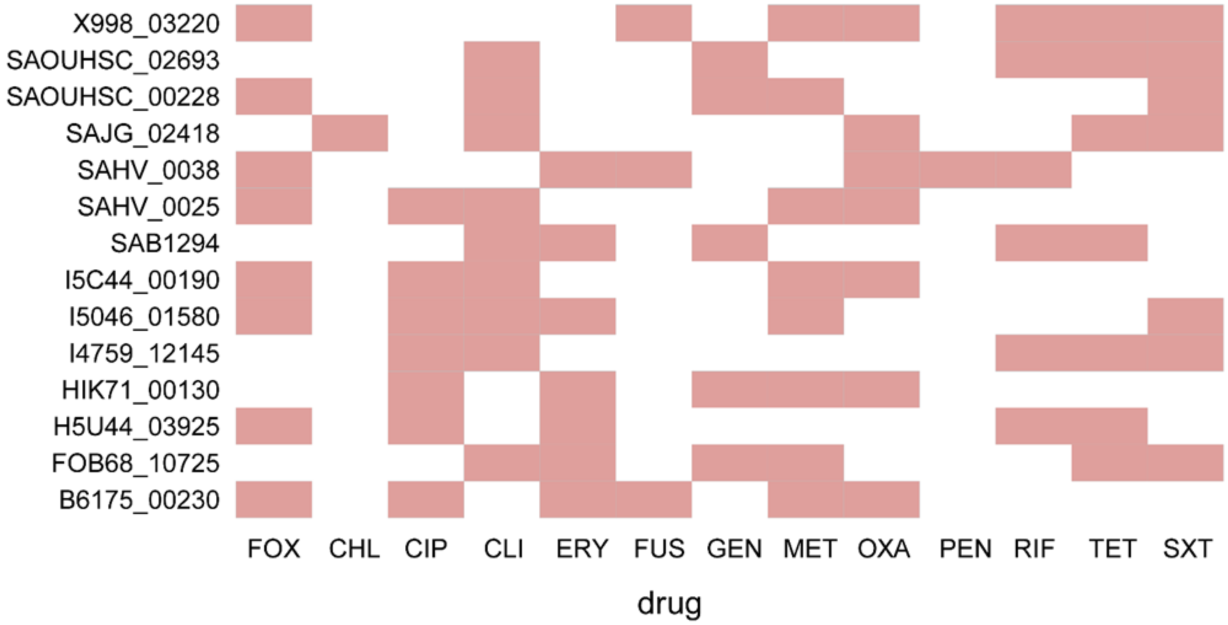

Supplementary Figure 1. Genomic map for discriminating antimicrobial resistance phenotypes across 13 antibiotics in *S. aureus* (Red markers represent a higher contribution of the gene to the differentiation of resistance/sensitivity to this antibiotic, while blanks indicate a lower contribution).
